# The meaning of being a visiting child of a seriously ill parent receiving care at the ICU

**DOI:** 10.1080/17482631.2021.1999884

**Published:** 2021-11-15

**Authors:** Susanne Knutsson, Marie Golsäter, Karin Enskär

**Affiliations:** a Child, School of Health and Welfare, Jönköping University, Jönköping, Sweden; b Faculty of Health and Life Sciences, Department of Health and Caring Sciences, Linnaeus University, Växjö, Sweden; c Child Health Services, Region Jönköping County, Jönköping, Sweden; and Department of Health, Medicine and Caring, Linköping University, Linköping, Sweden; d Department of Women´s and Children´s health, Malmö University, Malmö, Sweden

**Keywords:** Visiting, child, intensive care, information, caring

## Abstract

**Purpose:**

Children’s visits to the ICU are still restricted, and more focus on the child’s own needs and experiences are needed. The aim of this study is to illustrate the meaning of being a visiting child of a seriously ill parent receiving care at the ICU.

**Method:**

A qualitative descriptive design was used, with open-ended interviews with seven children (6–18 years) performed and analysed using a phenomenological research approach.

**Findings:**

Being a visiting child of a seriously ill parent receiving care at the ICU is described as a life situation taking place in an unfamiliar environment, characterized by a heartfelt, genuine desire to be there, in an interdependence entailing offering a loved one the help they need while at the same time being seen in a compassionate way and being able to share, revealing a sudden awakening of an inner truth of reality and a sense of a healing wisdom of understanding.

**Conclusions:**

The children felt good when they visited their ill parent, but at the same time not fully involved, and desired a more compassionate, caring approach by the nurses. Improvements are needed in how to approach visiting children in a more individual and caring way.

## Introduction

The topic of children’s visits to relatives being cared for at the intensive care unit (ICU) has been in focus for several years now (Ewens et al., 2021; Johnstone, 1994; Knutsson et al., 2004; Youngner et al., 1984), and a culture change around visitation policies and how to care for these children has taken place at many ICUs around the world. Visitor restrictions seem to have decreased as family members insist on access for their children (Hanley & Piazza, 2012), the media are highlighting family involvement (Hanley & Piazza, 2012), and research is showing evidence of benefits from children’s visits to the ICU, such as feelings of involvement (Hanley & Piazza, 2012; Knutsson et al., 2008; Knutsson & Bergbom, 2016, Knutsson et al. Knutsson, et al., 2017b) and diminished suffering (Knutsson et al., 2008; Knutsson & Bergbom, 2016, Knutsson et al. 2017b). However, nurses (Clarke, 2000, Knutsson & Bergbom 2007a) and parents still have their own (Knutsson & Bergbom 2007b) fear of the situation, along with an ambition to protect and care for both child and patient (Ewens et al., 2021). Nurses tend to rely on personal judgement rather than considering evidence or policy (Desai et al., 2020), and their attitudes, lack of skills in promoting emotional needs and in communicating with children (Ewens et al., 2021), as well as their educational level seem to have an impact on children visiting. Nurses with a higher education seem to show a greater awareness and implementation of professional values in their practice (Desai et al., 2020; Sibandze & Scafide, 2018). Organizational factors, including restrictive policies, age restrictions regarding the child, environmental factors, psychological trauma in the child, and infection risks to both child and patient, are also factors that still exist. The bond between parent and child is made vulnerable by the separation (MacEachnie et al. 2018), and needs to be maintained as the child expresses a need to be close to the ill parent and to be seen as a relative (Knutsson & Bergbom, 2016, MacEachnie et al. 2018). Therefore, there is a need to continuously highlight and increase the knowledge and awareness regarding the evidence promoting children’s visits and involvement (Ewens et al., 2021; Knutsson & Bergbom, 2016; Knutsson, Enskär, Golsäter et al., Knutsson, et al., 2017b; Knutsson et al., 2008; Vint, 2005).

To secure children’s rights in Sweden, an addition was made to legislation (*SFS 2010*) on 1 January 2010 (The patients safety act, 2010), stating that healthcare professionals have the responsibility to consider children’s need for information, advice, and support when a parent is seriously ill or unexpectedly dies. Along with this, the United Nations Convention on the Rights of the Child (UNCRC, , (2020)) entered into force in Sweden on 1 January 2020, highlighting children’s rights when it comes to involvement and information. The Convention’s Article 12 can be cited when it comes to children as relatives, asserting a child’s right to express their opinion and have it respected: “Parties shall assure to the child who is capable of forming his or her own views the right to express those views freely in all matters affecting the child, the views of the child being given due weight in accordance with the age and maturity of the child” (UNCRC, 2020:). Family support projects, including meetings with parents and children and age-appropriate information for children as next of kin and for their parents, have been tested and found to be helpful to both children and parents (Bugge et al., 2008, 2009; Golsäter et al., 2019, 2021). However, even though it has been proven that children of parents with a severe illness need information and support (MacEachnie et al. 2018, Eklund et al. 2020a, 2020b, 2020c, 2020d; Ewens et al., 2021; Golsäter et al., 2019; Golsäter et al., 2021; Knutsson et al. Knutsson, et al., 2017a, 2017b) and there is agreement regarding the need for a systematic approach (Andersson et al. 2018, Knutsson et al. 2017a, 2017b), no agreed-on programme for offering this can be found.

Additionally, a literature review (Järkestig Berggren & Hanson, 2013) involving 11 studies shows that research and knowledge regarding the child’s perspective when a parent is seriously ill have mainly been based on parents’ reports. The child’s perspective is lacking, and research (Ewens et al., 2021; Golsäter et al., 2021) concludes that children still experience being overlooked in their need for support, with the risk of being left in loneliness, sadness, and a lack of understanding of their parent´s situation. Some older studies on facilitating children’s visits at the ICU can be found (Baker et al., 1988; Clarke & Harrison, 2001; Johnson, 1994; Vint, 2005; Pierce 1998). But, there is a lack of newer and more contemporary studies adapted to current policies and laws related to children’s need to be involved using structured, child-centred interventions or frames, which can easily be used in clinical practice (Niemelä et al., 2010, MacEachnie, et al., 2018). There is also a lack of evaluations of the effect of information, advice, and support provided to children (Järkestig Berggren & Hanson, 2013). More focus on the child’s needs, feeling of involvement, and their own experiences regarding why the visit can be important for them is needed, and vital, in the work to secure children’s rights and diminish their suffering when a relative or a parent is seriously ill and receiving care at an ICU. As there is legislation (The patient safety act, 2010)SFS 2010, 1982:763 cap 5 §7) that regulates children’s rights when a parent is seriously ill, this study’s focus is on the parent, not the relative. The aim of this study is therefore to illustrate the meaning of being a visiting child of a seriously ill parent receiving care at the ICU.

## Materials and methods

### Design

A qualitative descriptive design was used in this study. Open-ended interviews were performed and analysed using Giorgi’s (2009) descriptive phenomenological research approach.

### Phenomenology

The descriptive phenomenological approach is a further development of Husserl’s phenomenology, with a close connection in the classical phenomenological theory formation (Giorgi, 2009). A phenomenological research approach focuses on a person’s individual lived experiences. The human body is seen as a subject, meaning that the body is full of memories and feelings that can create meaning. Everything a person experiences has a meaning, and originates in the lifeworld. In phenomenology there is a need for openness, flexibility, and sensitivity in order to do justice to the natural experience, and it is the experience of the overall structure of a phenomenon that is to be captured. The world should be described using as exact a description of the phenomenon as possible (Giorgi, 2009). According to Giorgi (2009), the researcher must start from the basic way of thinking about phenomenological reduction, the epoché of phenomenology. This means that the researcher scales away his preunderstanding and his idea of what already exists, in order to place all the focus on what actually reveals itself to consciousness. In other words, the researcher needs to approach the text with openness. According to Dahlberg et al. (2008), assuming a critically reflective attitude and bridling one’s prior understanding reduces the risk of the results being coloured by subjective opinions or judgements.

### Setting

An intensive care unit (ICU) at a county hospital in southern Sweden constitutes the setting for this study. According to Swedish law (The patient safety act, 2010), the unit has an ongoing process developing the work for children as relatives, and follows a clinical structure (Table I) for how to approach children as relatives, developed by the researchers in this study. The book *My Book—when Mom, Dad, or a sibling is sick* that is used in this approach was developed within the “child dialogue”, an arena for developing healthcare for children in the county. It is the child’s own book and the child determines its use, choosing whether to read it or to write or draw in it. The book is intended to be used when parents, staff, or others talk to the child about how they experience the situation and how they can get help in processing their thoughts and impressions. The approach is based on results of earlier studies in the research group (Golsäter et al., 2016; Knutsson & Bergbom, 2016, Knutsson et al. 2017a, 2017b), Knutsson’s earlier research within the area (Knutsson et al., 2004, Knutsson & Bergbom 2007a, 2007b, 2008; Knutsson & Bergbom, 2016), and experiences from the clinical intervention “See Me” (Golsäter et al., 2019, 2021) at an oncological unit in the county. “See Me” aims to facilitate the identification of and care for children as relatives, and to illuminate the child’s situation when a parent is seriously ill. It can be seen as a direct form of support for children and an indirect form of support for parents and ICU staff.

**Table I.** Overview of the clinical structure for how to approach children as relatives at the ICU

Stages	Actions to be taken by the nurse
*Identification*	Identify children as relatives as soon as possible after patient has been admitted
	Inform the parents about the child’s situation in having a seriously ill parent being cared for at the ICU
	Ask the parents to invite their child to visit the hospital and be given information about the sick parent Ask the parents if there are any children in the family, and encourage them by saying: At this unit we want the child to be a part of the ill parent’s situation by allowing them to visit and receive information. When do you think you can bring them with you?
*Encounter*	As soon as parents have given permission for the child to visit, offer a meeting at the unit for child and healthy parent together
	At the first meeting with child and healthy parent together, inform them about the sick parent’s illness and situation Encounter the child in a reflective, caring manner (Listen to their preunderstandings, be open to what they say, listen, see them, confirm, and be engaged, warm, and genuine. Think about the tone and body language you use). Encourage the child to ask questions in order to make them curious. Offer information about the parent’s treatment and disease based on the child’s previous experiences, age, and needs; and structure it according to guidelines, including both what to inform the child about and how. *Before* entering the patient’s room, offer information about what the patient and the surroundings look like. *During* the visit, be there to answer questions and explain how equipment works, etc.; and *after* the visit, ask if the child has more questions and tell them that if they think of any when they get home they can write them down and bring them next time. Offer the child a book for writing, drawing, and reflecting upon. Elaborate the child´s needs further.
	Offer the child an individual meeting with a nurse
	Offer child and healthy parent follow-up conversations to address the child’s needs for now and in the future
*Referral*	Offer child and healthy parent further support through one or two additional meetings with them in order to provide information and advice
	Facilitate referral for further expert support, such as hospital church, psychiatrist, or counsellor
	Facilitate referral to the child’s school nurse

### Participants

Participants were children (up to 18 years of age) of a seriously ill parent being treated at an ICU. The reason why the parent was receiving care at the ICU was sudden, unexpected, serious illness. All families (n = 5) with a seriously ill parent within a period of a year and a half were asked to participate in the study. Three families, including seven children (three boys and four girls aged 6–18 years [three being 6–12 and four being 13–18]), accepted participation. At the time of the interview, the patient had been receiving treatment at the unit for at least a week. All the parents survived the hospital visit.

The head of the unit was in charge of participant inclusion as she was working daytime five days a week and was considered to have an overview. Patients and their spouses and children (up to 18 years of age) were orally informed about the study by the head of the unit, with information received from the researcher and the information letter. The parents informed their children about the study, and if a child was interested in participating the parent informed the researcher, who then contacted and orally informed the child (or the parents, depending on the child’s age). At the time of the interview, parents and child received oral information about the study from the researcher and signed the consent form.

### Data collection

All children were interviewed about their experiences of being a visiting child of a seriously ill parent receiving care at the ICU. The informants chose both the time and place of the interviews, with five being held in the children’s home and two at the hospital. The three younger children (6–12 years) had their healthy parent or grandparent (one child) in the same room at the time of the interview, while the four older children were interviewed in a separate room. In order to stimulate recall and start the interview more naturally, the younger children (6–12 years) were asked to draw a picture of their sick parent in the hospital (Doverborg & Pramling Samuelsson, 2012) and the older children were asked to fill in a questionnaire about the nurses’ caring approach in the encounter with them (Caring Professional Scale, CPS) (Kalfoss & Owe, 2015; Swanson, 1991). This questionnaire consists of 15 items reflecting two aspects: *Competent Practitioner* (7 items) and *Compassionate Healer* (8 items). The interviews, conducted by the first author, began with the open question “Can you tell me about your experiences of visiting your seriously ill parent receiving care at the ICU?” Based on the child’s narratives, further questions were asked about how they had experienced the information, advice, and support they had received. The children were encouraged to speak as openly as possible, and follow-up questions such as “Can you describe further?” and “Can you tell me more?” were asked to obtain a clear and detailed description of the phenomenon (Kvale & Brinkmann, 2014). The interviews were conducted between August 2017 and December 2018, lasted 21–54 minutes (median 42 min), and were audio-recorded and transcribed verbatim.

### Data analysis

The analysis was carried out with a descriptive phenomenological research approach in accordance with Giorgi’s (2009) five principles. It began with several readings of the material by all three authors in order to gain a sense of the whole (1). Similar and different meaning units with focus on the phenomenon “the meaning of being a visiting child of a seriously ill parent receiving care at the ICU” were thereafter extracted from the text, as the goal of phenomenological analysis is to discern the meaning of the experience (2). Thereafter, transformation based on the scientific perspective was performed and the meaning in each meaning unit was described with the phenomenon in mind. For example, the first researcher asked herself “What does this meaning unit tell me about ‘the meaning of being a visiting child of a seriously ill parent receiving care at the ICU’, seen from a nursing/caring science perspective?” (3), and then described the meaning content as accurately as possible; this meant clarifying by describing the inherent meaning contained in the material. The researcher tried to clarify the implicit meanings and make explicit what was implicitly given. In accordance with Giorgi (2009) the transformation was kept at a descriptive level and did not go beyond what was directly given in the data, involving expanding rather than reducing the text. The text was checked regularly to verify that its transformation had not resulted in a loss of its original meaning. This is done in an attempt to vary the descriptions of a phenomenon until the most exact description of the meaning is reached (4). In order to achieve the general structure, the transformed text was reread several times and imaginary variations were used to distinguish between the phenomenon meaning one or more aspects—or constituents—of the studied object that are necessary for it to be able to form a whole. Free imaginative variation means that the researcher “thinks away” aspects from what is examined to ensure that this particular aspect is essential for the phenomenon to which it belongs; that it is the essence of the phenomenon. The general structure could thereafter be seen as a new whole, by understanding the meaning of the relationship between the constituents (5). The whole analysis process includes the application of the critical approach that the phenomenological reduction demonstrates, and also entails bridling one’s prior understanding, meaning that one’s own values or interpretations should not influence the analysis. The researcher needs to show an openness to and a compliance with data, and only talk about “findings” in terms of how the phenomenon presents itself. In this study, the results contain the general structure and three constituents. The two drawings (boy 6 years and boy 7 years) and four children’s (13–18 years) answers to the questions in the CPS that were used to stimulate the children’s talk are included in the results.

### Ethical consideration

Both written and oral information about the purpose of the study and how the interviews would be conducted was given to both parents and children before the interview. The written information letter and the oral information stated that participation was voluntary, that participants would be unidentified, and that the choice of whether or not to participate would not influence the parent’s forthcoming care at the unit. Both child and parent signed consent to participate in the study. The study followed the Helsinki Declaration (World Medical Association, 1964/2013), and was approved by the Ethical Committee at Linköping University: 2014/362-31.

## Findings

The essence of the phenomenon “being a visiting child of a seriously ill parent receiving care at the ICU” is described as a life situation taking place in an unfamiliar environment, characterized by a heartfelt, genuine desire to be there, in an interdependence entailing offering a loved one the help they need while at the same time being seen in a compassionate way and being able to share, revealing a sudden awakening of an inner truth of reality and a sense of a healing wisdom of understanding.

The phenomenon is revealed in perceptions of *being needed* and a need to help out with concrete things. The meaning of being needed is described as a mutual dependence revealed by the ill parent’s helplessness, and both the child’s and the parent’s need for closeness.

The need is concretized by being close to and present with the parent, as quickly and for as long a time as possible. The meaning of being needed is also expressed in a desire to ease the parent’s pain and an understanding that the parent needs help to recover, revealed in actions of wanting to help and naturally care for the parent. While visiting, the child has a desire to *be recognized* that manifests itself most strongly in the meeting with the nurse. The meaning of being recognized involves being seen, that someone initiated the visit, and being recognized through the nurse’s talks and questioning and through receiving information, but as this occurred only at a superficial, non-individual level the feeling of inclusion was not achieved. The children felt that the nurse cared for them, but not in a compassionate, caring way. The visit also revealed that the children had *become aware* of their sick parent’s situation by seeing the parent in the unfamiliar environment with all the equipment and medicines, and through drawings, books, and diaries. The children became aware of the shift in their parent’s condition, of the seriousness of the situation, and of how much they loved and longed for their parent. The meaning of becoming aware entailed seeing the reality and reaching understanding. All these feelings revealed a desire for a more compassionate, caring nurse who used a more genuine caring approach, involved the child in the situation on an individual basis, and included things the child experienced as important to them and their feelings of wellbeing.

### Being needed

Being needed is one of the constituents of the phenomenon at hand. The children saw being needed as a meaningful factor that influences their visit, and further described it in reference to their sick parent’s helplessness. The visit places the parent’s helplessness in focus, and even in the child’s uncertainty in seeing that their parent cannot perform by themselves, they help out. They described their desire to “be there” for their parent as meaningful and important. They also described helping their sick parent with various concrete things:

I helped Dad press the button for his bed

(Boy, 6 years)

The children also described closeness to their parent as meaningful, as they believed their presence could help the parent recover and return to them in their daily life. Being close to the parent created feelings of being significant and calmness in the child. The children described a desire to be close to their sick parent. They sat near their bed and looked at them; they did not always talk, but they were there. Sometimes they held their sick parent’s hand, and sometimes they talked a great deal with them as they felt that this helped their parent:

I held his hand

(Girl, 12 years)

The meaning of being needed is described as a state of mutual dependence revealed by the sick parent’s helplessness, and both the child’s and the parent’s need for closeness.

“Time” is also described as meaningful when it comes to the children’s feelings of being needed, and is seen in the dimensions of wanting to be with the parent as quickly as possible and wanting to stay there for a longer while. If “time” was extended, this caused worries and longing. The children could sit by their parent’s bed for hours, and often stayed almost a whole day. They felt good when they stayed with their parent. They also described that it had seemed like an eternity before they could see their parent because it took so long to drive there, and that the question about the children visiting had been brought up late:

Mother: We had waited several days before we brought the children to visit … … Child: Eight thousand years … Mother: Three days … Child: Eight million years … …

(Boy, 6 years)

The children described seeing their parent in pain, being sick. This was difficult for the children. However, they felt that seeing the parent and showing compassion gave the visit meaning and described it as meaningful; they wanted to help ease their parent’s pain and sickness. Showing compassion gave the children feelings of being helpful.

### Being recognized

The second constituent of the phenomenon is being recognized. The meaning of being recognized entailed being seen, that someone initiated the visit, and being talked to. The children further described that it was their healthy parent, or a physician or nurse, who had initiated their visit with their sick parent. It was typically the healthy parent who had to take the initiative. However, when the children were at the hospital they felt that the nurses recognized them in a nice way, approaching them, seeing them, and caring for them. They said hello, told the child their name, looked them in the eye, and talked a little. The children thought the nurses were nice and positive towards them, but felt that their approach was on a superficial level. This superficial approach is described as lacking a personal and emotional dimension, which can cause feelings of disclosure. The children experienced that the nurses were clinically competent and recognized them, but were not personally or emotionally involved and were not good listeners:

The nurses haven’t sat down and talked with me … … but they’ve talked a little

(Girl, 14 years)

The children expressed feelings of wanting the nurses to sit down and talk with them more, wanting them to see their feelings more. They wanted the nurses to be closer to them and more confirming, and wanted to be more involved in the situation.

The meaning of the visit also involved being recognized through questions and through receiving the book from the nurse. The children described that the nurses asked them questions: whether they had any questions, if they wanted a book to write in, or if they wanted ice cream. However, this was performed on a superficial level and could make them ambivalent and afraid to ask questions, and may therefore have had a negative impact on their understanding.

The children described the book itself as valuable and meaningful to them. They both wrote and drew in it. They described that they felt relaxed when the nurses asked if they wanted ice cream. However, the nurses did not ask questions about what they had written or drawn in the book they had been given, whether they wanted to know more about their sick parent’s condition, or how they felt:

No one sat down and talked about what was in the diary … … … a nurse simply came and asked if I had any questions … …

(Girl, 14 years)

The children expressed a longing for questions that helped them feel better and understand more.

They also described that the nurses did not see the person in front of them, did not see the individual child, and did not succeed in making the child curious and interested in knowing more, or in wanting more individualized information. They simply gave the child the information that was imposed on them. However, they described that they could have gotten more information before entering their sick parent’s room when it came to the environment, the machines, and their sick parent. They described wanting to be prepared, to know things in advance. The children wanted more of both oral and written information, and expressed disappointment at not receiving necessary information, for example, about the machines or the medicine. They described that when the nurses gave them information in the room, they did not understand, or relatively quickly forgot, what they were saying:

I could hardly understand what they were saying

(Boy, 10 years)

The meaning of being recognized also involved receiving information about the situation and the sick parent, and the children expressed a need for more comprehensive and personalized information; they felt that the information was given on a superficial level.

Sometimes the children did not get information from the nurse and needed to ask their healthy parent. However, the parent was not always able to answer the child, in which case the child asked them to ask the nurse. The children also described that they did not need more information from the nurse as their healthy parent had given them all the information they needed. While the children were technically informed, they described a further and deeper need for individual information.

The children described that it became obvious through the visit that their school needed to know about the situation involving their sick parent. It was either the nurse or the healthy parent who brought this up, and it was either the child and parent together or the parent alone who spoke with the child’s teacher. Recognizing that the school needs information indirectly acknowledges the child’s dependence on significant others and need for normal daily routines.

### Becoming aware

The third constituent of the phenomenon is becoming aware. The meaning of becoming aware is described as suddenly realizing something and reaching understanding. The children described that the book they had received from the staff to write in kept them occupied when they visited, but also at home where they would look at it. The children described a feeling of pride and affection for the book, and said it helped them be aware of their sick parent’s situation. The book became a meaningful link to the sick parent, a link the children did not want to let go of and that kept them aware that their parent was not present in person in their daily life at the moment:

This is MY book.

(Girl, 12 years)

The children described their book and showed what was in it. Their descriptions focused on the equipment in the sick parent’s room: machines, tubes, and medicines. The medicines their parent received through pumps and needles were in deep focus: They explained what the parent received, as well as why and how. The children described the medicines and the bed very specifically and thoroughly; these were important to them:

Drawing in here:

(Girl, 12 years)

The description of the medicines and the bed contributed to the children having become aware that their parent was dependent on them for survival. They continued talking about the medicines, and realized that they were important and that their parent was very sick as they needed so many of them.

The children also drew pictures of their parents in their book, and were eager to tell that *this is Mom* and *this is Dad*. The books contained descriptions and drawings of the cause of their sick parent’s present situation in a bed in an ICU:

And so it says: Name, Dad here. Fallen.

(Boy, 6 years)

The child’s visit helped them face reality, and realize how serious the situation was and how much they missed, needed, and loved their parent. The visit became a “reality-opener”.

The children described that when they had seen their sick parent in reality they saw how many needles, machines, and medicines were needed for them to get better. The equipment, bed, machines, medicines, tube in the throat, needles, blood hose, and urine catheter caught their attention. The fact that their parent could not urinate by themselves affected the children deeply in both an emotional and a humorous way. They also blamed their sick parent’s confusion on the medicines. Further, the children described that sometimes their parent was awake, while other times they slept through the whole visit. When seeing their sick parent, the child became aware that they were in pain and in a fragile position. They described this as tough, and said that seeing all this made them afraid. But at the same time, they described that it was good to see their sick parent and did not want to miss it. The child realized how sick their parent was upon seeing everything. The children also described that when they visited, they could see changes in their sick parent and in their room. They described that they noticed when there were more or fewer machines, hoses, and medicines. The parent’s situation and condition also made the children wonder about the future:

Will Mom be able to walk? To eat by herself? To be home with us?

(Girl, 15 years)

This awareness of their parent’s condition created feelings in the children such as sadness, anger, and fear concerning what would happen in the future.

The visits made the children aware of progress and regress in the patient’s situation. This awareness led to an understanding that their parent truly was sick, and of the severity of the sickness.

## Discussion

In this study, children described a feeling of being needed. They described a helplessness in watching their sick parent being in pain and being sick, and described this as a difficult experience. But even though this experience can be traumatic, it also enables children to better understand the reality and to maintain their relationships with family members (Lamiani et al., 2021). A loved one’s sickness affects the whole family’s health (Benzein et al., 2008), even that of the children. In this study, closeness to the parent, by sitting next to their bed, was interpreted as not only helping the child’s emotional state but also helping the parent to recover. Eriksson (2018) stresses human beings’ dependence on each other, adopting Lögstrup’s (1994) thoughts that each person lives in an inescapable situation of mutual dependence on other people. This interdependence is often obvious in families and is vital to recognize, as it is important for the experience of health (Eriksson, 2018). For children, the chance to reconnect with their parent reduced the sense of powerlessness and exclusion (Knutsson et al., 2007b). Sitting next to their parent’s bed gave the child the possibility to be engaged in their care process, which in this study was shown to be meaningful to the child and to make them feel important. A systematic review (Lamiani et al., 2021) of the psychological effects on children visiting family members at the ICU also shows the value of being close to and touching one’s family member. The encounter allows children to spend time with their parent and to demonstrate that they care (Lamiani et al., 2021). If the child’s emotions are not taken into account, feelings of suffering can arise; feeling significant and needed may reduce these feelings of suffering. Not being able to receive and give love involves boundless suffering (Eriksson, 2018). However, if the visit is not facilitated, children may be afraid to touch their family member or fear that they will harm them and thus stand at a distance, uncertain as to what to do (Knutsson & Bergbom, 2016).

The children in this study described a need to be recognized. Being seen and confirmed may diminish suffering (Eriksson, 2018). The children also described a need to be engaged in their parent’s care rather than simply being present. Eriksson and Granlund (2004) define participation as “a feeling of belonging and engagement experienced by the individual in relation to being active in a certain context”, divided into two dimensions—presence (i.e., physically being there) and engagement (i.e., expressions of involvement) (Granlund et al., 2012)—which is in line with what the children in this study desired, as they expressed a need to be involved in the care. Involvement, according to Imms et al. (2017), can be operationalized as actual engagement in the activity. Eklund et al. (2020c) interviewed children of parents in palliative care and found that they were not passive participants, as they developed strategies for coping with the situation and wanted to be involved. Still, the adults did not always consider the children’s voices. The same study also found that all children were listened to but that only a quarter of them reached the minimum point cited in Article 12 of the UNCRC, with their views being taken into account (Eklund et al. 2020c). Including children by giving them information about the illness and their relative’s situation, and letting them visit the relative at the hospital, are ways of promoting children’s participation in the relative’s and the family’s situation (Golsäter et al., 2019, 2021). Prerequisites for being involved in a parent’s care are early identification, information, and preparation, which are also important aspects for the child’s wellbeing and understanding (Knutsson & Bergbom, 2016, Knutsson et al. 2017b). Families experience an unmet need for information and support, which implies that healthcare professionals may want to acknowledge and include the family already at the time of diagnosis in order to help them endure and cope with the distressing experience and thus increase their wellbeing (Holst-Hansson et al., 2017).

Engaging children in their parent’s care requires that the children be given information about the illness and their parent’s situation. In this study the children were only given the information that was imposed, by the unit, on the nurses to give, and expressed a need for more information, especially before entering their sick parent’s room containing the environment, the machines, and the sick parent. Studies involving children of a parent with cancer show that a child having knowledge of what is happening to their sick parent during hospitalization is crucial to their wellbeing and relationships (Davey et al., 2012; Maynard et al., 2013). Children of a parent with cancer described having access to a nurse with knowledge about their parent’s specific situation as a prerequisite for getting the information they wanted (Golsäter et al., 2021).

In this study the children expressed a need for more comprehensive and personalized information, describing that the information they received was given on a superficial level. Nurses need to have knowledge in supporting children and to be skilled at being aware of whether and how a child has understood the information and the situation involving a sick parent. The nurses in Tafjord (2020) describe a need for more knowledge about children’s development in order to be able to talk to and inform them about a parent’s disease. When they lack knowledge, this can hinder them in approaching the children, resulting in the children being left alone. Golsäter et al. (2017) found that nurses’ self-confidence when meeting children as relatives must be increased through education in order to strengthen their professional role. The children in this study experienced that the nurses were clinically competent and recognized them, but were not personal or emotionally involved and were not good listeners. The children described the nurses as competent practitioners but not as compassionate healers (Swanson, 1991, 2000). They described a desire for a more sensitive manner and closer attention from the nurse. This was also found in the interviews in Golsäter et al. (2021) with children as relatives of a severely ill parent with an oncological disease receiving care at an oncological unit, showing a need for further improvements to nurses’ caring competencies, especially the aspects involving compassionate caring. Compassionate care constitutes the very essence, the core, of caring (Eriksson, 2018), and the outcome of experienced caring may depend on the nurse’s bearing (Koskinen et al., 2020).

This study’s findings also show that the child’s teachers and healthcare professionals at the school need to be contacted about the child’s situation. This is in line with the study by Golsäter et al. (2017), in which school nurses describe that they recognize the child through routine and collaboration, and through this contribute to making the school a safe place for the child during their parent’s illness trajectory.

The findings in this study show that, by visiting the ICU, the child becomes aware of the situation. The children understood the situation’s severeness when actually seeing their sick parent and the high-technological equipment in real life. Instead of fantasies, their thoughts became real and concrete, which is in congruence with Pierce’s (1998) findings. The children were also made more aware by writing in the book the nurses had given them. Doverborg and Pramling Samuelsson (2000) suggest that it is facilitating to let a child write and explain what they are writing, as they can then associate it to something that exists in their experience. This awareness, the reality-opener, may lead to knowledge and understanding (Gadamer, 2004).

**Figure 1.**
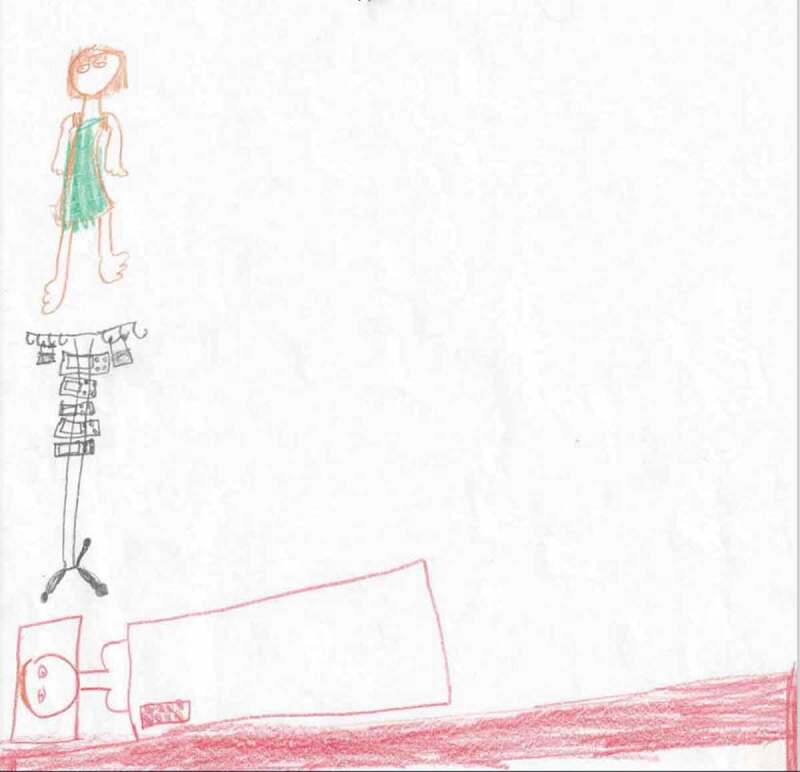
Child’s drawing of a nurse, the equipment, and their parent in a bed

When a family member is sick, the family pursues balance by attempting to hold together and at the same time maintain a positive attitude while battling fear and treatment-related side-effects (Holst-Hansson et al., 2017). In this study, the children described this as a need to be present at the ICU. To date, interventions to support children as next of kin to a sick parent have mainly been family interventions (Järkestig Berggren & Hanson, 2013), showing good results on the parent’s situation. In a study on supporting families through a family intervention in an oncological context in Sweden, the families reported that the intervention was positive and a unique kind of family health conversation, which had afforded them the opportunity to communicate and share their experiences as a family group (Holst-Hansson et al., 2020). Another family intervention with children as next of kin has been shown to meet both the ill parent’s and the healthy co-parent’s expectations (Alvariza et al., 2021). In same intervention, most of the participating children appreciated the structure and content of the intervention and felt seen, heard, and acknowledged (Eklund et al., 2020a). Still, family interventions risk highlighting the adults’ needs and wishes while not fully prioritizing the children’s needs (Eklund et al., 2020b, 2020c). The approach used in this study had a clearer focus on the children (see Figure 1), and was found to be very well received by the participating children both in this study and in an oncological context (Golsäter et al., 2021). Therefore, we suggest that future interventions for children as relatives of a parent with a severe physical disease offer information, advice, and support based mainly on a child-centred approach rather than merely a family-centred one (Coyne et al., 2018).

## Discussion of the Method

The study used a qualitative design based on interviews. It was found that the research question matched the method, which matched both the data and analytical procedure; thus, methodological coherence was obtained. Hearing children’s views is important and necessary (Doverborg & Pramling Samuelsson, 2012; Golsäter et al., 2021), and may offer new possibilities for insights into a child’s perspective on visiting a parent at the ICU. The interview with each child was unique, and the focus was kept on the children’s experiences of visiting their parent at the ICU in both the data-gathering and the analysis process (Kortesluoma et al., 2003).

Research involving children is not without its methodological challenges (Kirk, 2007). In a phenomenological analysis it is not the individual structures that should emerge but the general ones, in order to clarify the phenomenon and make it transferable to other contexts. A phenomenon is illustrated through its depth; it is not a matter of how many people have experienced the same thing (Giorgi, 2009). While the experiences of seven children can be regarded as a limitation, this was found to be sufficient for gathering material containing rich meaning (Giorgi, 2009) and for allowing in-depth analysis of the data (Sandelowski (2000). But, it is possible that the children who chose not to participate had different experiences from those of the study’s participants. The analysis was performed by all three researchers individually to increase comprehension, soundness of the data interpretation, and consistency (Polit & Beck, 2016). The authors’ natural attitudes regarding the phenomenon were discussed, and one’s own values or interpretations, according to Giorgi (2009), were considered in order to avoid influencing the analysis.

Another limitation of the study is the age span of the participating children, 6–18 years, as cognitive development may differ between a 6-year-old and an 18-year-old. However, there are other factors that are equally likely to affect children’s experiences and ability to respond to interview questions (Kortesluoma et al., 2003). Interviews with children are often short, which can be a problem in the analysis. However, in this study we tried to make it easier for the children to sustain their concentration so they would be more likely to answer the questions (Kortesluoma et al., 2003), by talking a bit about something else prior to the interview and letting them draw (Doverborg & Pramling Samuelsson, 2012). This is why the children’s descriptions connected to the drawings and the answers to the questions in the CPS, which were used to stimulate the children’s talk, are included in the results. All the researchers are nurses, and have previously worked with and talked to children about what they think and how they feel about healthcare. However, there is always a risk of re-traumatizing a child when recalling their experiences. This was accounted for by letting the parents and the child know that if the child needed support afterwards they were welcome to contact the first researcher, who would forward the contact to an appropriate source of support such as a counsellor. None of the children or parents requested such support.

## Clinical implications

In order to improve care for children as relatives, nurses could meet regularly in reflection groups to discuss and highlight:

- how to involve children as relatives of a parent receiving care at the ICU. The use of a clinical structure for how to approach children as relatives can be useful and valuable to both staff, parents, and child.
- the need for awareness and knowledge about the importance of identifying, welcoming, and involving the child at an early stage in their seriously ill parent’s situation.
- the need to see the child and care for them with a compassionate, caring approach. Education about compassionate caring or caritative caring could be useful to nurses.

- the need to be aware that their bearing may have an impact on children’s experiences and their ability to diminish suffering. Education about the impact of one’s bearing and about what is caring and un-caring could be useful.
- the need to give the child individualized support and information about their ill parent and the parent’s situation. Education about the impact of involvement could be useful.
- the need for child-friendly resources, such as a book or diary to write in and/or referral to further sources of support, such as a child life specialist, school nurse, or counsellor.

## Conclusion

The frame used in this study may have a positive influence on children’s knowledge and participation in their parent’s care. The findings show that the children felt welcome and needed at the hospital, felt recognized by the nurses, and became aware of the severeness of their parent’s condition and situation, but needed information, knowledge, and involvement in their parent’s situation; all this based on their individual situation and needs. The children felt good when visiting, but at the same time desired a more compassionate, caring approach by the nurses. The approach therefore needs a supplement describing the importance of approaching the child in a more individual and caring way. There is a need for more research on how to involve children in these situations and how to approach children in a caring way, and on the impact of nurses’ bearing on their ability to diminish suffering when it comes to children as visitors at the ICU.
